# Pro-Inflammatory Cytokine IL-1β Up-Regulates CXC Chemokine Receptor 4 via Notch and ERK Signaling Pathways in Tongue Squamous Cell Carcinoma

**DOI:** 10.1371/journal.pone.0132677

**Published:** 2015-07-15

**Authors:** Yi Sun, Demao Zhu, Guihua Wang, Di Wang, Huashan Zhou, Xueting Liu, Manli Jiang, Lingjuan Liao, Zhiguang Zhou, Jinyue Hu

**Affiliations:** 1 Department of Pathology, The Second Xiangya Hospital, Central South University, Changsha, China; 2 Medical Research Center, Changsha Central Hospital, Changsha, China; 3 Department of Pathology, Changsha Central Hospital, Changsha, China; 4 Department of Oncology, Changsha Central Hospital, Changsha, China; 5 Institute of Metabolism and Endocrinology, The Second Xiangya Hospital, Central South University, Changsha, China; University of San Francisco, UNITED STATES

## Abstract

Chronic inflammation contributes to tumor development through the induction of oncogenic mutations, genomic instability, early tumor promotion, and enhanced angiogenesis. Here, we report that IL-1 receptor 1 (IL-1R1) was expressed in 40 of 41 human tongue squamous cell carcinomas (TSCC). IL-1β up-regulated the expression of CXCR4, a CXC chemokine receptor that mediates cancer growth and metastasis, at both mRNA and protein levels in Tca8113 TSCC cells. IL-1β treatment of Tca8113 cells promoted migration in response to CXCR4 ligand stromal-derived factor α (SDF-1α). The inhibition of IL-1R1 by its antagonist IL-1Ra or RNA interference significantly reversed the up-regulation of CXCR4 induced by IL-1β. IL-1R1 activation also up-regulated the expression of IL-1β itself, suggesting a positive feedback regulation of CXCR4 expression. Furthermore, IL-1β induced the activation of Notch, which was originally considered a stem cell regulator. Pharmacological inhibition of Notch signaling reversed the up-regulation of CXCR4 induced by IL-1β, suggesting that Notch signaling may be involved in the growth and metastasis of cancers via up-regulation of CXCR4. In addition, IL-1β induced the activation of extracellular signal regulated kinase (ERK) and ERK inhibition decreased the up-regulation of CXCR4 induced by IL-1β, suggesting the involvement of ERK signaling in cancer metastasis. Taken together these data suggest that IL-1β and IL-1R1 promote cancer growth and metastasis by up-regulating CXCR4 expression and that CXCR4 may be a link between inflammation and cancer.

## Introduction

Inflammatory responses play diverse roles at different stages of tumor development, including initiation, promotion, malignant conversion, invasion, and metastasis [1]. Inflammation caused by bacterial or viral infections increases cancer risk [2]. Chronic Helicobacter pylori infection is associated with gastric cancer [3] and mucosa-associated lymphoid tissue lymphoma [4, 5]. Infections with hepatitis B or C viruses increase the risk of hepatocellular carcinoma [6]. Infection with Schistosoma is linked to bladder cancer [7], and infection with bacteroides species is linked to colorectal cancer [8]. Infection with Epstein-Barr Virus is associated with nasopharyngeal carcinoma [9] and Burkitt lymphoma [10]. Finally, tobacco smoking promotes tumor development in part by triggering chronic inflammation [11].

IL-1β is a pleiotropic pro-inflammatory cytokine that has profound effects on inflammation and immunity. Polymorphisms of IL-1β, IL-1 receptor 1 (IL-R1), or IL-1 receptor antagonist (IL-1Ra) are associated with an increased risk of various solid malignant tumors, including gastric cancer [12], pancreatic cancer [13], lung cancer [14], prostate cancer [15], and breast cancer [16]. Human carriers of IL-1B polymorphisms (IL-1B-511T and IL-1B-31C) show enhanced IL-1β production and increased circulating levels of the cytokine, resulting in an increased risk of cancers [17]. IL-1 mRNA is highly expressed in more than half of all tested metastatic human tumor specimens, including non-small-cell lung carcinoma, colorectal adenocarcinoma, and melanoma [18]. Stomach-specific expression of human IL-1β in transgenic mice leads to spontaneous gastric inflammation and cancer that correlates with early recruitment of myeloid-derived suppressor cells (MDSCs) to the stomach [19]. However, the detailed mechanisms explaining the effect of IL-1β on cancer development are not fully understood.

Chemokines, small pro-inflammatory chemoattractant cytokines, were originally identified as mediators of leukocyte trafficking and homing. Chemokines bind to specific G-protein-coupled seven trans-membrane chemokine receptors [20]. The chemokine CXCL12 (stromal-derived factor-1, SDF-1) binds primarily to CXC receptor 4 (CXCR4, CD184), which is also an HIV co-receptor [21]. CXCR4 is expressed on lymphocytes, hematopoietic stem cells, endothelial and epithelial cells, as well as multiple types of cancer cells, including breast cancer, ovarian cancer, prostate cancer pancreatic cancer, melanoma, esophageal cancer, lung cancer, bladder cancer, osteosarcoma, neuroblastoma, leukemia, gastric cancer, and nasopharyngeal carcinoma [22, 23]. The CXCL12 and CXCR4 axis is involved in tumor progression, angiogenesis, metastasis, and survival [24]. A wide variety of potential drugs targeting CXCL12/CXCR4 and downstream signaling pathways, including peptides, small molecules, antibodies, and small interfering RNA, have been tested for cancer therapy [24].

CXCR4 is expressed in multiple types of cancer. Hypoxia is a prominent regulator of CXCR4 via HIF-1α [25], and inhibition of HIF-1α decreases the metastasis of cancers [26]. The pro-inflammatory cytokines TNF-α and IL-1β are also involved in the regulation of CXCR4 in human astroglioma cells [27], suggesting that inflammation may promote cancer development via CXCR4. Here, we report that IL-1R1 is widely expressed in clinical tongue squamous cell cancer tissues. IL-1β induces the up-regulation of CXCR4 in the tongue carcinoma cell line Tca8113, suggesting that CXCR4 is a link between inflammation and cancer.

## Materials and Methods

### Cell lines and reagents

Tca8113 is a tongue squamous cell carcinoma cell line [28]. Hep2 is a human laryngeal carcinoma cell line [29,30]. All cells were grown in DMEM containing 10% FCS, 100 units/ml penicillin, and 100 mg/ml streptomycin. Recombinant human IL-1β, IL-1Ra, and mouse anti-human CXCR4 antibody (FACS) were purchased from R&D systems (Minneapolis, MN). Rabbit anti-human CXCR4 polyclonal antibody (western blot) was purchased from Abcam (Cambridge, MA). Notch inhibitor L685458 was purchased from Sigma-Aldrich (St. Louis, MO). Rabbit anti-human Notch1 antibody, rabbit anti-human phosphorylated ERK, JNK, and p38 antibodies, rabbit anti-human total ERK, JNK, p38, and β-actin antibodies, and ERK inhibitor U0126 were purchased from Cell Signaling Technology (Beverly, MA). Human IL-1R1 shRNA plasmid and control shRNA were purchased from Santa Cruz Biotechnology (Santa Cruz, CA, USA).

### Immunohistochemical analysis

Human tissue specimens were approved for use without informed consent by the Ethics Committee of Changsha Central Hospital. Primary tongue squamous cell carcinoma tissue from routine diagnostic biopsy specimens were obtained from the Department of Pathology, the Second Xiangya Hospital of Central South University, and the Department of Pathology, Changsha Central Hospital. Tissues were fixed with 4% formaldehyde, embedded in paraffin, and sectioned. Endogenous peroxidase activity was blocked by hydrogen peroxide (0.3%). Slides were stained with IL-1R1 antibodies (1:50), followed by incubation with a biotinylated anti-mouse secondary antibody (1:1000) and horseradish peroxidase–conjugated streptavidin. Color was developed with diaminobenzidine, and sections were counterstained with hematoxylin.

### Reverse transcription-PCR (RT-PCR)

Total RNA was extracted from 1 to 2 × 10^6^ cells using TRIzol (Invitrogen, Carlsbad, CA, USA), as described by the manufacturer. mRNA was reverse transcribed with RevertAid (MBI Fermentas, Burlington Ontario, Canada) at 42°C for 60 min, and the resulting cDNA was subjected to PCR (94°C for 1 min followed by 20–25 cycles at 94°C for 30 s, 60°C for 30 s, and 68°C for 1 min and an extension for 10 min at 68°C). The PCR products were separated on 1.0% agarose gels and visualized with ethidium bromide. The forward and reverse primer pairs were (5΄ to 3΄) as follows:

β-actin-F, TCGTGCGTGACATTAAGGAGA,

β-actin-R, ATACTCCTGCTTGCTGATCCA;

GAPDH-F, AATCCCATCACCATCTTCCA,

GAPDH-R, CCTGCTTCACCACCTTCTTG;

IL-1β-F, TGAACTGAAAGCTCTCCACCT,

IL-1β-R, ACTGGGCAGACTCAAATTCCA;

IL1R1-F, TGCCTGCTTGAAGGAACAGT

IL1R1-R, ATTCTTGGTCATCATCACCCC

IL1R2-F, TCCTGACATTTGCCCATGAA

IL1R2-R, TTCTGAATATTCCTGGCGTG

CXCR4-F, TGACTTGTGGGTGGTTGTGTT

CXCR4-R, TCGGTGATGGAAATCCACTT

CCR6-F, ATCGTAATGAAGTTGGGGTT

CCR6-R, ATCACAAATTTCAGACCCCT

CCR7-F, ACTCCATCATTTGTTTCGTG

CCR7-R, TAGTATCCAGATGCCCACAC

### Immunoblot

Cells (1–2 × 10^6^) were lysed in 200 ml lysis buffer (20 mM Tris, pH 7.5, 150 mM NaCl, 1% Triton X-100, 1 mM EDTA, 1 mM sodium pyrophosphate, 1 mM β-glycerophosphate, 1 mM Na_3_VO_4_, 1 mg/ml leupeptin). The cell lysate was centrifuged at 12,000 g at 4°C for 5 min. Proteins were electrophoresed on 10% SDS-PAGE gels, and transferred onto Immobilon P membranes (Millipore, Billerica, MA, USA). The membranes were blocked by incubation in 3% nonfat dry milk for 1 h at room temperature and then incubated with primary antibodies (1:1000) in PBS containing 0.01% Tween 20 at 4°C overnight. After incubation with a horseradish peroxidase-conjugated secondary antibody (1:2000), the protein bands were detected with SuperSignal chemiluminescent substrate-stable peroxide solution (Pierce Rockford, IL, USA) and BIOMAX-MR film (Eastman Kodak Co., Rochester, NY, USA). When necessary, the membranes were stripped with Restore Western blot stripping buffer (Pierce) and re-probed with antibodies against various cellular proteins.

### Quantitative real time RT-PCR (qRT-PCR)

qRT-PCR was performed as described by Sun *et al*. [31]. Briefly, total RNA from the cells was isolated and reverse transcribed as described above. The cDNA was amplified using TaqMan Universal PCR master mix (Applied Biosystems, Foster City, CA, USA) and an ABI Prism 7500 sequence detection system (Applied Biosystems). The amplification of the target genes was normalized using the amplification levels of glyceraldehyde-3-phosphate dehydrogenase (*GAPDH*) as an endogenous control. The efficiency of the PCR was tested by amplification of the target from serially diluted cDNA generated by reverse transcription of a stock set of human RNA. The data analysis and calculations were performed using the 2^−ΔΔ*CT*^ comparative method, as described by the manufacturer. Gene expression is shown as the fold induction of a gene in IL-1β-treated samples relative to samples cultured with control medium.

### Flow cytometry analysis

For CXCR4 protein detection, Tca8113 cells were cultured for 24 h in 6-well plates, harvested and washed with fluorescence-activated cell sorting (FACS) buffer (5 mM EDTA, 0.1% NaN3, and 1% FCS, in Dulbecco’s phosphate-buffered saline (PBS)). After incubation with a monoclonal antibody against human CXCR4 for 40 min on ice, the cells were stained with a FITC-labeled secondary antibody and examined for protein expression by flow cytometry (BD Biosciences).

### Transwell migration assays

In vitro cell migration assays were performed using Transwell chambers with polyethylene terephthalate membrane (24-well inserts, 8.0 μm, Corning). Briefly, 2.5 × 10^4^ cells, treated with medium or IL-1β for 24 h, were seeded onto the upper chambers. Medium containing 10% FBS with 20 ng/ml SDF-1α was placed in the lower chamber to serve as a chemoattractant. Twenty-four hours later, the cells on the upper surface of the filter were removed by gently wiping with a cotton swab. The cells that migrated to the lower surface of the filter were fixed with 95% alcohol for 5 min and incubated with a mixture of 50 μg/ml PI (Sigma–Aldrich) and 25 mg/ml RNase A (Sigma–Aldrich) at 37°C for 30 min. Migrated cells were visualized by fluorescence microscopy (Leica, Wetzlar, Germany). Cells in three fields per filter were counted under the microscope.

### shRNA plasmid transfection

Cells were cultured in six-well plates and transfected with 1 μg of a plasmid containing hairpin RNA targeting human interleukin-1 receptor 1 (IL-1R1) or with 1 μg of a control plasmid containing non-specific hairpin RNA with Lipofectamine 2000 (Invitrogen) according to the manufacturer’s instructions. Expression of IL-1R1 in the transfected cells was examined by western blot 48 h after transfection. For stable transfection, G418-resistant cells were selected after incubation with 800 μg/ml G418 for 3 weeks.

### Statistical analysis

All experiments were performed at least three times, and representative results are shown. The results are expressed as the mean ± S.D. Differences between groups were examined for statistical significance using Student’s *t* test, and *P* values equal to or less than 0.05 were considered statistically significant (n = 3 for each qRT-PCR test).

## Results

### Tongue squamous cell carcinomas express IL-1 receptor 1

**Fig 1:**
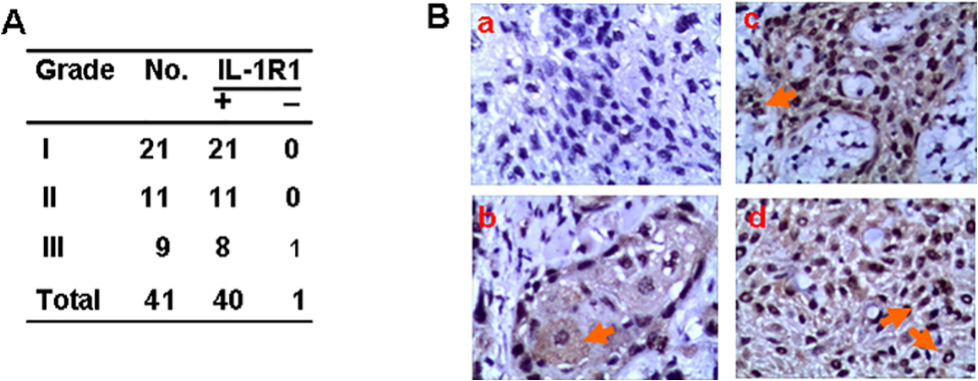
The expression of IL-1R1 in human tongue squamous cell carcinoma (TSCC). (A) IL-1R1 expression in human TSCC. Biopsies of human primary TSCC were obtained during routine diagnostic procedures. Specimens were sectioned, stained with anti-human IL-1R1 antibody, and counterstained with hematoxylin. (B) Representative negative staining in poorly differentiated TSCC (Ba), and representative positive staining in well (Bb), moderately (Bc), and poorly (Bd) differentiated TSCC. The arrow in Figure Bb indicates the positive cytoplasmic staining. The arrow in Figure Bc indicates the positive nuclear staining in stromal cells. The arrows in Figure Bd indicate positive nuclear and peri-nuclear staining.

We first detected IL-1R1 protein expression in biopsy specimens of human tongue squamous cell carcinomas. Sections from 40 of 41 tongue carcinoma tissues stained positively for IL-1R1 protein by immunohistochemistry (Fig 1A and 1B). Eight of 9 poorly differentiated tongue carcinomas (grade III), all 11 moderately differentiated tongue carcinomas (grade II), and all 21 well differentiated tongue carcinomas (grade I), were IL-1R1 positive (Fig 1A). When we observed the staining patterns of IL-1R1, we found that tongue squamous cell carcinomas were positive in both the cytoplasm and nucleus (Fig 1B). In poorly differentiated carcinomas, peri-nuclear staining was observed (Fig 1B). Little membrane staining was observed (Fig 1B). In addition, some positive nuclear staining was also observed in stromal cells. These results indicated that IL-1R1 is widely expressed in tongue squamous cell carcinoma.

### IL-1β up-regulates CXCR4 expression

**Fig 2:**
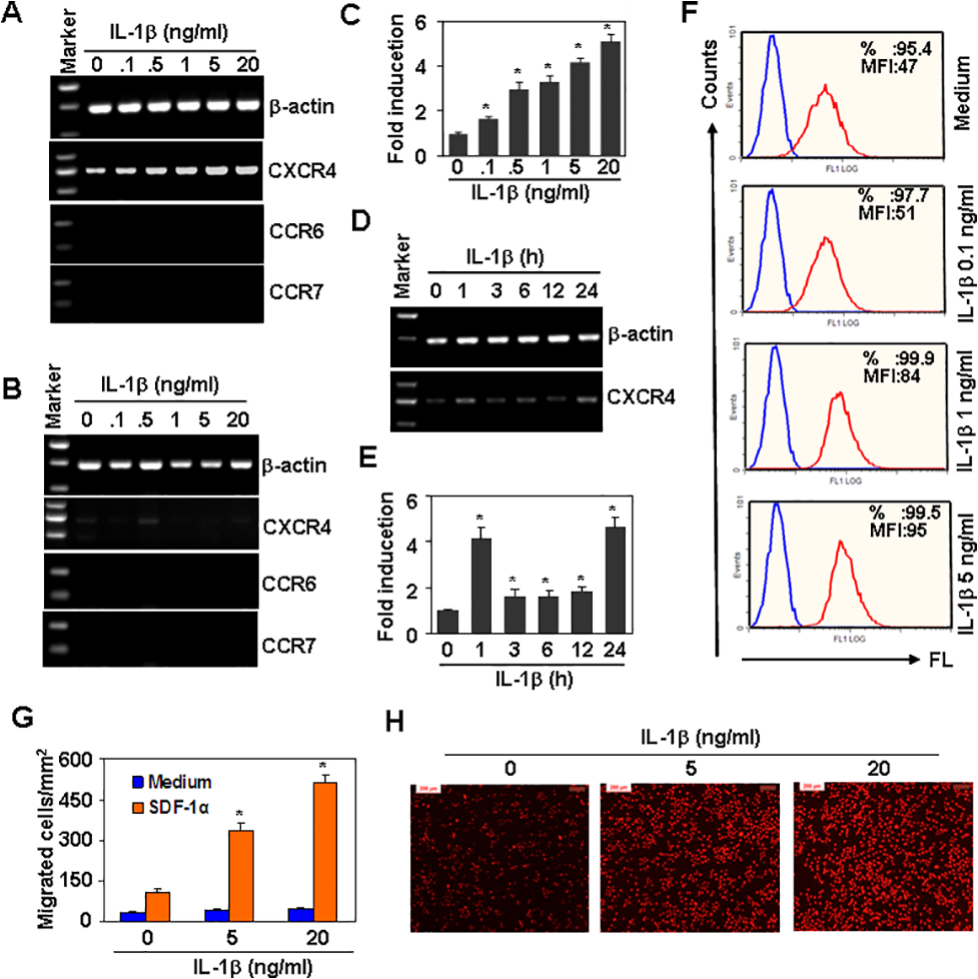
IL-1β up-regulates CXCR4 expression. (A&B) The effect of IL-1β on the mRNA expression of chemokine receptors in Tca8113 (A) and Hep2 (B) cells. Cells were treated with 20 ng/ml IL-1β for 24 h. The mRNA levels of CXCR4, CCR6 and CCR7 were measured by RT-PCR. β-actin mRNA levels were measured as loading controls. (C) Quantitative CXCR4 mRNA expression in (A). * *P* < 0.05 compared with the non-treated group. (D) Time course of CXCR4 mRNA expression in response to IL-1β stimulation. Tca8113 cells were treated with 20 ng/ml IL-1β for the indicated time periods. The mRNA levels of CXCR4 were detected by RT-PCR. β-actin mRNA levels were measured as loading controls. (E) The quantitative data corresponding to (D). * *P* < 0.05 compared with the non-treated group. (F) The effect of IL-1β on CXCR4 protein expression. Tca8113 cells were treated with the indicated concentrations of IL-1β for 24 h. CXCR4 protein expression was detected by FACS. (G) The effect of IL-1β on SDF-1α-induced cell migration. Tca8113 cells were treated with the indicated concentrations of IL-1β for 24 h. Cell migration in response to medium or 20 ng/ml SDF-1α was measured by the Transwell assay. *P < 0.05 compared with control groups. (I) The transwell assay showed cell migration in response to 20 ng/ml SDF-1α after treatment with the indicated concentrations of IL-1β for 24 h (Scale bars: 200 μM).

IL-1β has been reported to up-regulate CXCR4 expression in astroglioma cells [27]. We measured the effect of IL-1β on CXCR4 expression in human tongue squamous cell carcinoma Tca8113 cells. IL-1β upregulated the levels of CXCR4 transcripts in a dose-dependent manner (Fig 2A). However, IL-1β did not up-regulate CXCR4 mRNA levels in human laryngeal epidemoid carcinoma Hep2 cells (Fig 2B). IL-1β did not regulate CCR6 or CCR7, two other CC chemokine receptors related to cancer metastasis [32,33], in either Tca8113 or Hep2 cells (Fig 2A and 2B). Quantitative real-time RT-PCR showed that the up-regulation of CXCR4 transcript induced by IL-1β was significant in Tca8113 cells (Fig 2C). IL-1β-induced up-regulation of CXCR4 was also time-dependent (Fig 2D and 2E). Two peaks were found, with one peak at 1 h and another peak at 24 h (Fig 2D and 2E). FACS results showed that IL-1β up-regulated CXCR4 protein levels in a dose-dependent manner (Fig 2F). Transwell assay results showed that IL-1β dose-dependently promoted cell migration in response to the CXCR4 ligand SDF-1α (Fig 2G and 2H)

### IL-1RI is responsible for the induction of CXCR4

**Fig 3:**
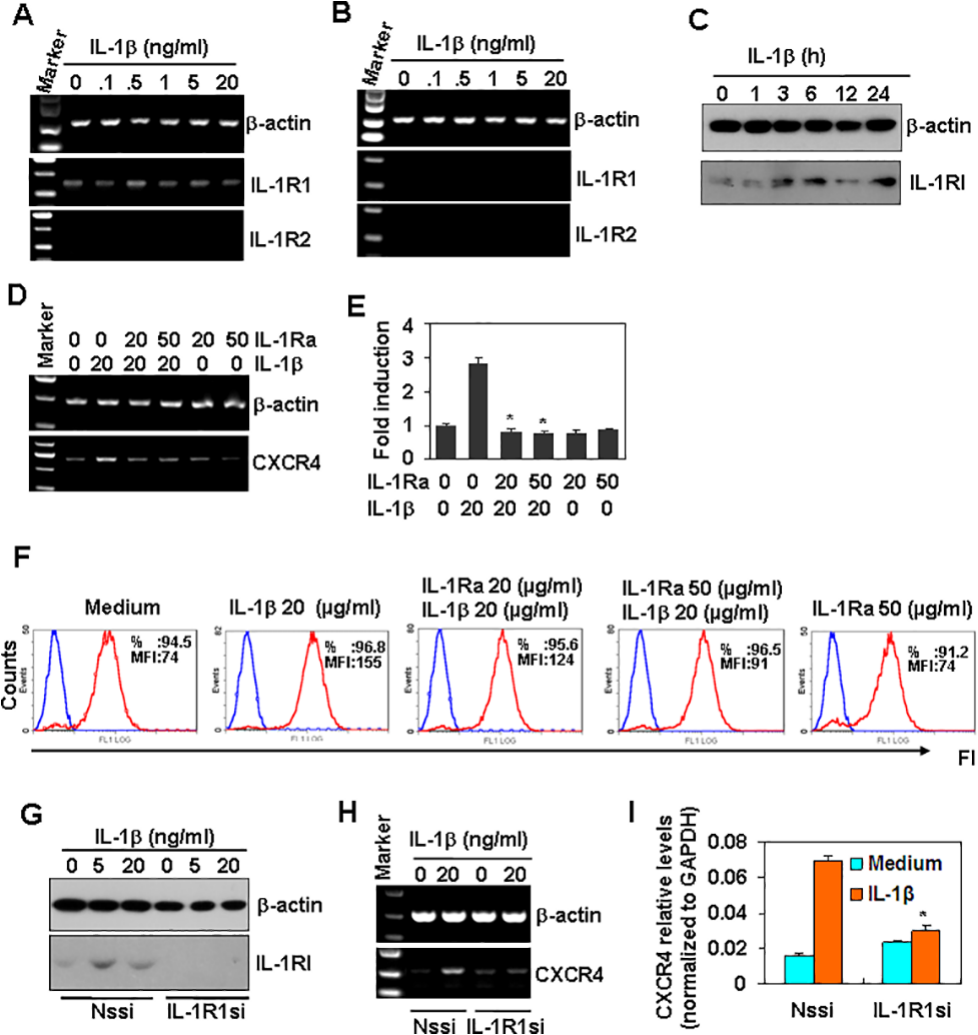
IL-1RI is responsible for the induction of CXCR4. (A) The expression of IL-1 receptors in Tca8113 cells. Cells were treated with the indicated concentrations of IL-1β for 24 h. The mRNA levels of IL-1R1 and IL-1RII were measured by RT-PCR. β-actin mRNA levels were measured as loading controls. (B) The expression of IL-1 receptors in Hep2. Cells were treated as described in (A). The mRNA levels of IL-1R1 and IL-1R2 were measured by RT-PCR. β-actin mRNA levels were measured as loading controls. (C) The effect of IL-1β on IL-1R1 expression. Tca8113 cells were treated with the indicated concentrations of IL-1β for 24 h. The protein levels of IL-1R1 were measured by western blot. β-actin protein levels were measured as loading controls. (D&E) The effect of IL-1Ra on IL-1β-induced CXCR4 mRNA up-regulation. Tca8113 cells, pre-treated with the indicated concentrations of IL-1Ra for 1 h, were treated with or without 20 ng/ml IL-1β for 1 h. The mRNA levels of CXCR4 were measured by RT-PCR (D) and qRT-PCR (E). * *P* < 0.05 compared with the IL-1β-treated group. (F) The effect of IL-1Ra on IL-1β-induced CXCR4 protein up-regulation. Tca8113 cells, pre-treated with the indicated concentrations of IL-1Ra for 1 h, were treated with or without 20 ng/ml IL-1β for 24 h. The protein expression of CXCR4 was measured by FACS. (G) The effect of RNA interference on the expression of IL-1R1 protein. Tca8113 cells, transfected with non-specific shRNA (Nssi) or with IL-1R1 shRNA (IL-1R1si), were treated with the indicated concentrations of IL-1β for 24 h. The expression of IL-1R1 protein was measured by western blot. β-actin protein levels were measured as loading controls. (H) The effect of IL-1R1 down-regulation on CXCR4 mRNA expression. Non-specific shRNA (Nssi) or IL-1R1 shRNA (IL-1R1si) transfected Tca8113 cells were treated with medium or 20 ng/ml IL-1β for 24. The mRNA levels of CXCR4 were measured by RT-PCR. β-actin mRNA levels were measured as loading controls. (I) The quantitative data corresponding to (H). * *P* < 0.05 compared with the Nssi group.

To determine why IL-1β induced the up-regulation of CXCR4 in Tca8113 cells but not in Hep2 cells, we measured the expression of IL-R1 and 2 in these cell lines. RT-PCR results showed that Tca8113 cells expressed IL-1R1 but not IL-1R2 (Fig 3A). However, Hep2 cells expressed neither IL-1R1 nor 2 (Fig 3B). In addition, western blot results showed that treatment of Tca8113 with IL-1β up-regulated the protein levels of IL-1R1 (Fig 3C). These results suggest that the expression of IL-1R1 was responsible for the induction of CXCR4 induced by IL-1β. As IL-1Ra is a strong antagonist of IL-1R1, we tested the effect of IL-1Ra on the induction of CXCR4 induced by IL-1β. IL-1Ra pre-treatment of Tca8113 cells significantly inhibited the up-regulation of CXCR4 induced by IL-1β at both the mRNA and protein levels (Fig 3D, 3E and 3F). In addition, when IL-1R1 was down-regulated by RNA interference (Fig 3G), the IL-1β-induced up-regulation of CXCR4 was prevented (Fig 3H and 3I).

### Positive feedback regulation of IL-1β and CXCR4 expression

**Fig 4:**
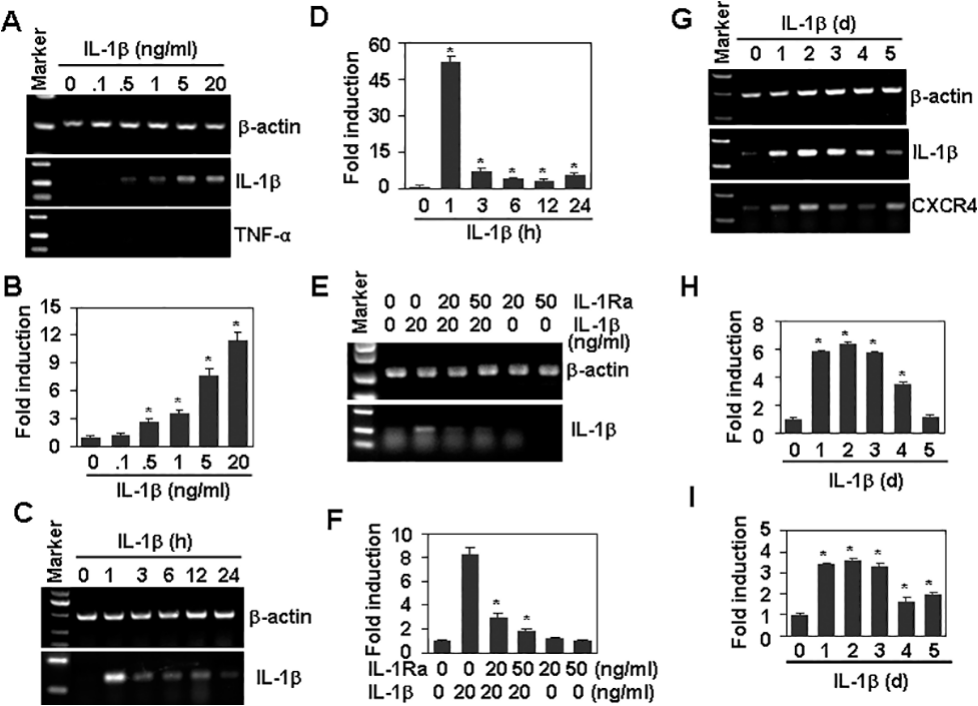
The positive feedback regulation of IL-1β and CXCR4 expression. (A) The effect of IL-1β on mRNA levels of IL-1β and TNF-α. Tca8113 cells were treated with the indicated concentrations of IL-1β for 24 h. The mRNA levels of IL-1β and TNF-α were measured by RT-PCR. β-actin mRNA levels were measured as loading controls. (B) IL-1β quantitative mRNA levels in (A). * *P* < 0.05 compared with the non-treated control. (C) Time-course of IL-1β mRNA expression in response to IL-1β treatment. Tca8113 cells were treated with 20 ng/ml IL-1β for the indicated time periods. The mRNA levels of IL-1β were measured by RT-PCR. β-actin mRNA levels were measured as loading controls. (D) IL-1β quantitative mRNA levels in (C). * *P* < 0.05 compared with the non-treated group. (E) The effect of IL-1Ra on IL-1β-induced IL-1β mRNA expression. Tca8113 cells, pre-treated with the indicated concentrations of IL-1Ra for 1 h, were stimulated with 20 ng/ml IL-1β for 24 h. The mRNA levels of IL-1β were measured by RT-PCR. β-actin mRNA levels were measured as loading controls. (F) IL-1β quantitative mRNA levels in (E). * *P* < 0.05 compared with the non-treated group. (G) The sustained effect of IL-1β on the expression of IL-1β and CXCR4. Tca8113 cells were treated with 20 ng/ml IL-1β for the indicated number of days. The mRNA levels of IL-1β and CXCR4 were measured by RT-PCR. β-actin mRNA levels were measured as loading controls. (H-I) Quantitative data of IL-1β (H) and CXCR4 (I) in (G). * *P* < 0.05 compared with the non-treated groups.

An IL-1β-mediated positive-feedback loop has been reported to play a protective role against Clostridium difficile infection [34]. In this study, we found that IL-1β treatment of Tca8113 induced a significant up-regulation of IL-1β in a dose- and time-dependent manner at the transcript level (Fig 4A, 4B, 4C and 4D). As a control, IL-1β did not up-regulate the expression of TNF-α (Fig 4A). Up-regulation of IL-1β transcript was also dependent on IL-1R1, as IL-1Ra pre-treatment also inhibited the up-regulation of IL-1β (Fig 4E and 4F). This IL-1β positive feedback loop induced a sustained up-regulation of both IL-1β and CXCR4. IL-1β and CXCR4 up-regulation persisted for 4 days after a single treatment of Tca8113 cells with IL-1β (Fig 4G, 4H and 4I).

### The Notch signaling pathway is involved in the up-regulation of CXCR4 induced by IL-1β

**Fig 5:**
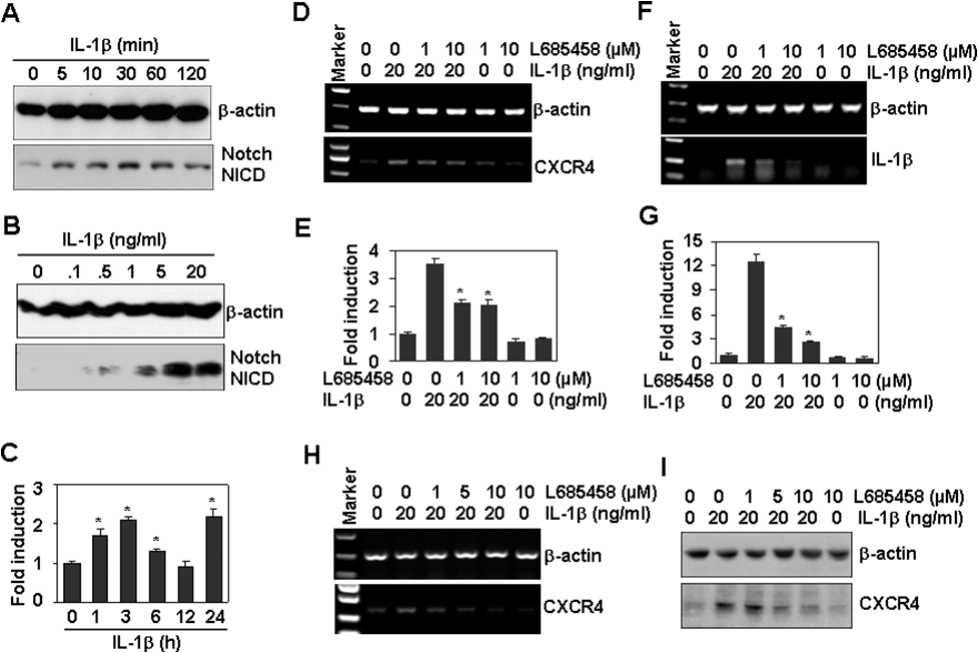
The Notch signaling pathway is involved in the up-regulation of CXCR4 induced by IL-1β. (A) Time-dependent activation of Notch by IL-1β. Tca8113 cells were treated with 20 ng/ml IL-1β for the indicated time periods. Activated Notch NCID fragments were detected by western blot. β-actin protein levels were measured as loading controls. (B) Dose dependent activation of Notch by IL-1β treatment for 1 h. (C) The effect of IL-1β on Hes1 mRNA levels. Tca8113 cells were treated with 20 ng/ml IL-1β for the indicated time periods. The mRNA levels of the Notch1 targeting gene Hes1 were measured by qRT-PCR. * *P* < 0.05 compared with the control group. (D) The effect of Notch inhibition on CXCR4 expression induced by IL-1β. Tca8113 cells, pre-treated with the indicated concentrations of Notch inhibitor L685458 for 30 min, were treated with 20 ng/ml IL-1β for 1 h. The mRNA levels of CXCR4 were measured by RT-PCR. β-actin mRNA levels were measured as loading controls. (E) Quantitative data of CXCR4 expression in (D). * *P* < 0.05 compared with IL-1β-treated alone group. (F) The effect of Notch inhibition on IL-1β expression induced by IL-1β. Cells were treated as described in (D). The mRNA levels of IL-1β were measured by RT-PCR. β-actin mRNA levels were measured as loading controls. (G) Quantitative data of IL-1β expression in (F). * *P* < 0.05 compared with the IL-1β-treated alone group. (H) The effect of Notch inhibition on long-term CXCR4 mRNA expression induced by IL-1β. Tca8113 cells, pre-treated with the indicated concentrations of Notch inhibitor L685458 for 30 min, were treated with 20 ng/ml IL-1β for 24 h. The mRNA levels of CXCR4 were measured by RT-PCR. β-actin mRNA levels were measured as loading controls. (I) The effect of Notch inhibition on CXCR4 protein expression induced by IL-1β. Tca8113 cells, pre-treated with the indicated concentrations of the Notch inhibitor L685458 for 30 min, were treated with 20 ng/ml IL-1β for 24 h. The protein levels of CXCR4 were measured by western blot. β-actin protein levels were measured as loading controls.

Notch signaling has been reported to modulate CXCR4 expression [35]. To determine whether the observed IL-1β-induced CXCR4 up-regulation was related to the activation of Notch signaling, we first measured the activation of Notch1 induced by IL-1β in Tca8113 cells. Western blot results showed that treatment with IL-1β induced the cleavage of Notch1 to form the activated fragment NICD (Notch intracellular domain) in a dose- and time-dependent manner (Fig 5A and 5B). RT-PCR showed that IL-1β treatment up-regulated the mRNA levels of the Notch-targeting gene Hes1 mildly but significantly (Fig 5C). These results suggest that IL-1β activates Notch signaling [36]. Moreover, Notch inhibition by the inhibitor L685458 decreased the up-regulation of CXCR4 induced by 1 h of IL-1β treatment (Fig 5D and 5E). Notch inhibition also decreased the induction of IL-1β transcript induced by IL-1β (Fig 5F and 5G). To determine the effect of Notch inhibition on the long-term expression of CXCR4 mRNA, Tca8113 cells were pre-treated with L685458 for 30 min and then treated with IL-1β for 24 h. RT-PCR showed that Notch inhibition reversed the IL-1β-induced up-regulation of CXCR4 mRNA (Fig 5H). Western blot results also showed that Notch inhibition reversed IL-1β-induced CXCR4 protein up-regulation (Fig 5I). These results demonstrate that the Notch pathway is involved in the induction of CXCR4 induced by IL-1β.

### IL-1β-induced ERK activation is involved in the up-regulation of CXCR4 expression

**Fig 6:**
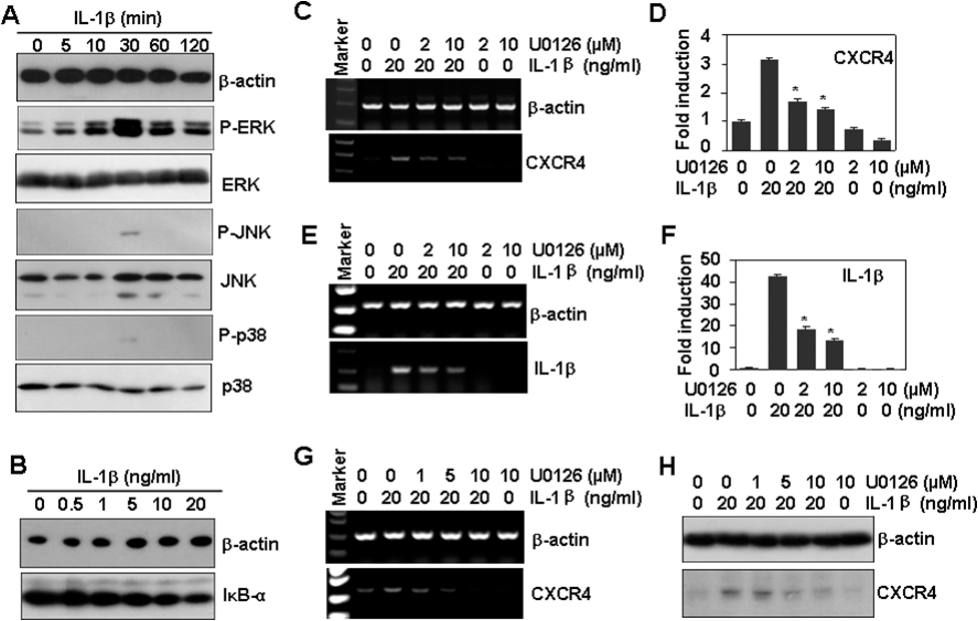
IL-1β-induced ERK activation is involved in the up-regulation of CXCR4 expression. (A) The effect of IL-1β on the activation of MAPKs. Tca8113 cells were treated with 20 ng/ml IL-1β for the indicated time periods. The phosphorylation levels of MAPKs were measured by western blot. β-actin protein levels were measured as loading controls. (B) The effect of IL-1β on the activation of IκB-α. Tca8113 cells were treated as described in (A). IκB-α protein levels were measured by western blot. β-actin protein levels were measured as loading controls. (C) The effect of ERK inhibition on IL-1β-induced CXCR4 expression. Tca8113 cells, pre-treated with the indicated concentrations of U0126 for 30 min, were stimulated with 20 ng/ml IL-1β for 1 h. The mRNA levels of CXCR4 were measured by RT-PCR. β-actin mRNA levels were measured as loading controls. (D) Quantitative data of (C). * *P* < 0.05 compared with the IL-1β-treated group. (E) The effect of ERK inhibition on IL-1β-induced IL-1β mRNA expression. Tca8113 cells were treated as described in (C). The mRNA levels of IL-1β were measured by RT-PCR. β-actin mRNA levels were measured as loading controls. (F) Quantitative data of (E). * *P* < 0.05 compared with the IL-1β-treated group. (G) The effect of ERK inhibition on long-term CXCR4 mRNA expression induced by IL-1β. Tca8113 cells, pre-treated with the indicated concentrations of ERK inhibitor U0126 for 30 min, were treated with 20 ng/ml IL-1β for 24 h. The mRNA levels of CXCR4 were measured by RT-PCR. β-actin mRNA levels were measured as loading controls. (I) The effect of ERK inhibition on CXCR4 protein expression induced by IL-1β. Tca8113 cells, pre-treated with the indicated concentrations of ERK inhibitor U0126 for 30 min, were treated with 20 ng/ml IL-1β for 24 h. The protein levels of CXCR4 were measured by western blot. β-actin protein levels were measured as loading controls.

In response to IL-1β binding to IL1R1, a complex sequence of combinatorial phosphorylation and ubiquitination events results in activation of the nuclear factor NF-κB and MAPK signaling pathways, which induce the expression of IL-1β target genes [37]. We measured the activation of MAPKs, including ERK, JNK and p38, induced by the treatment of Tca8113 cells with IL-1β. Western blot results showed that IL-1β significantly induced phosphorylation of ERK and slightly induced the activation of JNK and p38 in a time-dependent manner (Fig 6A). IL-1β had little effect on the activation of NF-κB or the degradation of IκB-α (Fig 6B). We therefore focused on ERK signaling and measured the effect of ERK inhibition on IL-1β-induced CXCR4 up-regulation. RT-PCR results showed that the inhibition of ERK by the specific inhibitor U0126 significantly reversed the up-regulation of both CXCR4 (Fig 6C and 6D) and IL-1β (Fig 6E and 6F) induced by IL-1β treatment for 1 h. To determine the effect of ERK inhibition on the long-term expression of CXCR4 mRNA, Tca8113 cells were pre-treated with U0126 for 30 min and then treated with IL-1β for 24 h. RT-PCR showed that ERK inhibition also reversed the IL-1β-induced up-regulation of CXCR4 mRNA (Fig 6G). Western blot results also showed that ERK inhibition reversed IL-1β-induced CXCR4 protein up-regulation (Fig 6H). These results demonstrate that the ERK signaling pathway is also involved in the up-regulation of CXCR4 induced by IL-1β.

## Discussion

Oral tongue squamous cell carcinoma is one of the most prevalent tumors of the head and neck region. Despite advances in treatment, the survival of patients has not significantly improved over the past several decades. Human papillomavirus infection has been reported to be related to the pathogenesis of tongue carcinoma [38–40]. Neutrophils, CD8+ and CD4+ lymphocytes infiltrate tongue carcinomas, and infiltrating immune cells produce pro-inflammatory cytokines [41, 42]. The pathogen recognition receptor toll-like receptor 5 has been reported to play an important role in the pathophysiology of tongue carcinoma and might represent a link between bacterial infection and cancer [43]. This suggests that tongue squamous cell carcinoma is inflammation-related cancer.

IL-1β is up-regulated in solid cancers, including breast, colon, lung, head and neck cancers, and melanomas. Patients with IL-1β-producing tumors have generally bad prognoses [44]. IL-1α and IL-1β transcript levels have been evaluated in melanoma, non-small cell carcinoma, colon, and squamous cell cancer cell lines via real-time qRT-PCR, and several of these cell lines exhibit significantly increased levels of IL-1α or IL-1β [18]. IL-1 exhibits autocrine behavior by stimulating the tumor cell to invade and proliferate or exerts a paracrine effect on stromal cells in the microenvironment [44]. In this study, we did not detect the expression of IL-1β in resting Tca8113 cells, but treatment with IL-1β induced an up-regulation of IL-1β in a dose- and time-dependent manner, suggesting that IL-1β exhibits autocrine behavior in Tca8113 cells.

Metastasis is the major factor that limits survival in cancer patients. The chemokine receptor CXCR4 promotes cancer metastasis in breast cancer and melanoma [45]. CXCR4 is also expressed in many types of cancer, and plays important roles in cancer metastasis [22]. CXCR4 is also related to cancer growth and proliferation [22]. HIF-1α is a major factor that promotes CXCR4 expression [25], and other factors may also regulate CXCR4 expression. In our study, we found that the activation of IL-1R1 significantly up-regulated CXCR4 levels. We have also found that another pro-inflammatory cytokine, tumor necrosis factor (TNF) α, up-regulated CXCR4 expression at both transcript and protein levels in nasopharyngeal carcinoma and colorectal cancer (data not shown). These studies suggest that pro-inflammatory cytokines may function to promote cancer growth, proliferation and metastasis via up-regulation of CXCR4.

Target-based therapies are widely considered to be the future of cancer treatment. The evidence that ERK signaling promotes cell proliferation, cell survival and metastasis, along with the high frequency at which this pathway is aberrantly activated in cancer, has led many labs to focus on developing ERK inhibitors for cancer therapy [46]. Tumor fibroblast-derived epiregulin, a 46-amino acid protein that belongs to the epidermal growth factor family, promotes the growth of colitis-associated neoplasms through ERK [47]. Periostin, an extracellular matrix protein that functions in tooth and bone remodeling, is produced by cancer-associated fibroblasts and supports growth of gastric cancer through the activation of ERK [48]. The blockade of ERK signaling inhibits tumor growth in various types of cancer, including gastric cancer [49], renal cell carcinoma [50], and rhabdomyosarcoma [51]. Conversely, ERK activation facilitates cancer invasion and metastasis [52, 53], and the inhibition of ERK signaling inhibits metastasis [50]. In our study, we found that ERK signaling is involved in the up-regulation of CXCR4 induced by IL-1β. The inhibition of ERK signaling prevented the up-regulation of CXCR4 induced by IL-1β. These results suggest that ERK may be a target for the prevention and control of tongue squamous cell carcinoma.

The Notch pathway is an evolutionarily conserved signaling system that regulates proliferation, differentiation, cell fate determination, and self-renewal of stem and progenitor cells in both embryonic and adult organs [54]. Notch signaling is a double-edged sword in cancer, as it can be either oncogenic or tumor suppressive, depending on the cellular context [55]. On the one hand, it is well established that Notch acts as an onco-protein in T-cell acute lymphoblastic leukemia/lymphoma (T-ALL), an aggressive tumor that occurs mainly in children and adolescents [55]. Notch signaling is also related to growth, invasion, and metastasis in other solid tumors, such as melanoma [56], glioma [57], breast cancer [58], and lung adenocarcinoma [59]. On the other hand, Notch signaling can function as a tumor suppressor. In conditional Notch1 knockout mice, an increased incidence in skin cancers has been reported, and the incidence of papilloma formation in Notch1 null skin was markedly increased by exposure to chemical carcinogens plus phorbol ester [60]. The expression of a specific inhibitor of the Notch transcription complex, dominant negative MAML1 (DN-MAML), in murine skin led to the development of cutaneous squamous cell carcinoma [61]. Several studies have reported conflicting results concerning Notch regulation of CXCR4 expression. Notch signaling has been reported to positively control CXCR4/SDF-1 expression and functions in myeloma cell lines, and forced CXCR4 activation partially rescues tumor cells from the effects of Notch inhibition [62]. By up-regulating CXCR4, Notch signaling is involved in lipopolysaccharide-induced maturation of bone marrow-derived dendritic cells [63]. In our study, we found that Notch is involved in IL-1β-induced CXCR4 up-regulation. Inhibition of Notch signaling decreased CXCR4 expression. However, the inhibition of Notch signaling by a γ-secretase inhibitor or knockout of the down-stream transcription factor RBP-J has been reported to significantly increase cell surface, total protein, and mRNA levels of CXCR4 in mesenchymal stem cells [64]. In endothelial cells, the expression of the Notch ligand Dll4 inhibited attachment and migration in response to stromal-derived growth factor 1 (SDF1) due to the down-regulation of CXCR4 [65]. Thus, the regulation of CXCR4 in response to Notch signaling seems to be cell type dependent.

## References

[1] Grivennikov SI, Greten FR, Karin M (2010) Immunity, inflammation, and cancer. Cell 140: 883–899. 10.1016/j.cell.2010.01.025 20303878 PMC2866629

[2] Clifford G, Franceschi S (2007) Immunity, infection, and cancer. Lancet 370: 6–7. 17617251 10.1016/S0140-6736(07)61023-X

[3] Polk DB, Peek RM Jr. (2010) Helicobacter pylori: gastric cancer and beyond. Nat Rev Cancer 10: 403–414. 10.1038/nrc2857 20495574 PMC2957472

[4] Nakamura S, Matsumoto T (2013) Helicobacter pylori and gastric mucosa-associated lymphoid tissue lymphoma: recent progress in pathogenesis and management. World J Gastroenterol 19: 8181–8187. 10.3748/wjg.v19.i45.8181 24363507 PMC3857439

[5] Wundisch T, Dieckhoff P, Greene B, Thiede C, Wilhelm C, Stolte M, et al (2012) Second cancers and residual disease in patients treated for gastric mucosa-associated lymphoid tissue lymphoma by Helicobacter pylori eradication and followed for 10 years. Gastroenterology 143: 936–942; quiz e913-934. 10.1053/j.gastro.2012.06.035 22750463

[6] Kew MC (2013) Hepatitis viruses (other than hepatitis B and C viruses) as causes of hepatocellular carcinoma: an update. J Viral Hepat 20: 149–157. 10.1111/jvh.12043 23383653

[7] Zaghloul MS, Nouh A, Moneer M, El-Baradie M, Nazmy M, Younis A (2008) Time-trend in epidemiological and pathological features of schistosoma-associated bladder cancer. J Egypt Natl Canc Inst 20: 168–174. 20029473

[8] Toprak NU, Yagci A, Gulluoglu BM, Akin ML, Demirkalem P, Celenk T, et al (2006) A possible role of Bacteroides fragilis enterotoxin in the aetiology of colorectal cancer. Clin Microbiol Infect 12: 782–786. 16842574 10.1111/j.1469-0691.2006.01494.x

[9] Lo KW, To KF, Huang DP (2004) Focus on nasopharyngeal carcinoma. Cancer Cell 5: 423–428. 15144950 10.1016/s1535-6108(04)00119-9

[10] Gromminger S, Mautner J, Bornkamm GW (2012) Burkitt lymphoma: the role of Epstein-Barr virus revisited. Br J Haematol 156: 719–729. 22482131 10.1111/j.1365-2141.2011.09007.x

[11] Takahashi H, Ogata H, Nishigaki R, Broide DH, Karin M (2010) Tobacco smoke promotes lung tumorigenesis by triggering IKKbeta- and JNK1-dependent inflammation. Cancer Cell 17: 89–97. 10.1016/j.ccr.2009.12.008 20129250 PMC2818776

[12] El-Omar EM, Carrington M, Chow WH, McColl KE, Bream JH, Young HA, et al (2000) Interleukin-1 polymorphisms associated with increased risk of gastric cancer. Nature 404: 398–402. 10746728 10.1038/35006081

[13] Barber MD, Powell JJ, Lynch SF, Fearon KC, Ross JA (2000) A polymorphism of the interleukin-1 beta gene influences survival in pancreatic cancer. Br J Cancer 83: 1443–1447. 11076651 10.1054/bjoc.2000.1479 PMC2363418

[14] Hu Z, Shao M, Chen Y, Zhou J, Qian J, Xu L, et al (2006) Allele 2 of the interleukin-1 receptor antagonist gene (IL1RN*2) is associated with a decreased risk of primary lung cancer. Cancer Lett 236: 269–275. 16019127 10.1016/j.canlet.2005.05.015

[15] Lindmark F, Zheng SL, Wiklund F, Balter KA, Sun J, Chang B, et al (2005) Interleukin-1 receptor antagonist haplotype associated with prostate cancer risk. Br J Cancer 93: 493–497. 16106254 10.1038/sj.bjc.6602729 PMC2361575

[16] Hefler LA, Grimm C, Lantzsch T, Lampe D, Leodolter S, Koelbl H, et al (2005) Interleukin-1 and interleukin-6 gene polymorphisms and the risk of breast cancer in caucasian women. Clin Cancer Res 11: 5718–5721. 16115908 10.1158/1078-0432.CCR-05-0001

[17] Fox JG, Wang TC (2007) Inflammation, atrophy, and gastric cancer. J Clin Invest 117: 60–69. 17200707 10.1172/JCI30111 PMC1716216

[18] Elaraj DM, Weinreich DM, Varghese S, Puhlmann M, Hewitt SM, Carroll NM, et al (2006) The role of interleukin 1 in growth and metastasis of human cancer xenografts. Clin Cancer Res 12: 1088–1096. 16489061 10.1158/1078-0432.CCR-05-1603

[19] Tu S, Bhagat G, Cui G, Takaishi S, Kurt-Jones EA, Rickman B, et al (2008) Overexpression of interleukin-1beta induces gastric inflammation and cancer and mobilizes myeloid-derived suppressor cells in mice. Cancer Cell 14: 408–419. 10.1016/j.ccr.2008.10.011 18977329 PMC2586894

[20] Zlotnik A, Yoshie O (2012) The chemokine superfamily revisited. Immunity 36: 705–716. 10.1016/j.immuni.2012.05.008 22633458 PMC3396424

[21] Didigu CA, Wilen CB, Wang J, Duong J, Secreto AJ, Danet-Desnoyers GA, et al (2014) Simultaneous zinc-finger nuclease editing of the HIV coreceptors ccr5 and cxcr4 protects CD4+ T cells from HIV-1 infection. Blood 123: 61–69. 10.1182/blood-2013-08-521229 24162716 PMC3879906

[22] Hu J, Deng X, Bian X, Li G, Tong Y, Li Y, et al (2005) The expression of functional chemokine receptor CXCR4 is associated with the metastatic potential of human nasopharyngeal carcinoma. Clin Cancer Res 11: 4658–4665. 16000558 10.1158/1078-0432.CCR-04-1798

[23] Zlotnik A, Burkhardt AM, Homey B (2011) Homeostatic chemokine receptors and organ-specific metastasis. Nat Rev Immunol 11: 597–606. 10.1038/nri3049 21866172

[24] Teicher BA, Fricker SP (2010) CXCL12 (SDF-1)/CXCR4 pathway in cancer. Clin Cancer Res 16: 2927–2931. 10.1158/1078-0432.CCR-09-2329 20484021

[25] Guo M, Cai C, Zhao G, Qiu X, Zhao H, Ma Q, et al (2014) Hypoxia Promotes Migration and Induces CXCR4 Expression via HIF-1alpha Activation in Human Osteosarcoma. PLoS One 9: e90518 10.1371/journal.pone.0090518 24618817 PMC3949690

[26] Staller P, Sulitkova J, Lisztwan J, Moch H, Oakeley EJ, Krek W (2003) Chemokine receptor CXCR4 downregulated by von Hippel-Lindau tumour suppressor pVHL. Nature 425: 307–311. 13679920 10.1038/nature01874

[27] Oh JW, Drabik K, Kutsch O, Choi C, Tousson A, Benveniste EN (2001) CXC chemokine receptor 4 expression and function in human astroglioma cells. J Immunol 166: 2695–2704. 11160334 10.4049/jimmunol.166.4.2695

[28] Yu T, Liu K, Wu Y, Fan J, Chen J, Li C, et al (2014) MicroRNA-9 inhibits the proliferation of oral squamous cell carcinoma cells by suppressing expression of CXCR4 via the Wnt/beta-catenin signaling pathway. Oncogene. 33: 5017–5027. 10.1038/onc.2013.448 24141785

[29] Brozovic A, Vukovic L, Polancac DS, Arany I, Koberle B, Fritz G, et al (2013) Endoplasmic reticulum stress is involved in the response of human laryngeal carcinoma cells to Carboplatin but is absent in Carboplatin-resistant cells. PLoS One 8: e76397 10.1371/journal.pone.0076397 24086737 PMC3781097

[30] Lee JH, Jeong YJ, Lee SW, Kim D, Oh SJ, Lim HS, et al (2010) EGCG induces apoptosis in human laryngeal epidermoid carcinoma Hep2 cells via mitochondria with the release of apoptosis-inducing factor and endonuclease G. Cancer Lett 290: 68–75. 10.1016/j.canlet.2009.08.027 19781850

[31] Sun R, Zhang Y, Lv Q, Liu B, Jin M, Zhang W, et al (2011) Toll-like receptor 3 (TLR3) induces apoptosis via death receptors and mitochondria by up-regulating the transactivating p63 isoform alpha (TAP63alpha). J Biol Chem 286: 15918–15928. 10.1074/jbc.M110.178798 21367858 PMC3091201

[32] Ito M, Teshima K, Ikeda S, Kitadate A, Watanabe A, Nara M, et al (2014) MicroRNA-150 inhibits tumor invasion and metastasis by targeting the chemokine receptor CCR6, in advanced cutaneous T-cell lymphoma. Blood 123: 1499–1511. 10.1182/blood-2013-09-527739 24385540

[33] Kochetkova M, Kumar S, McColl SR (2009) Chemokine receptors CXCR4 and CCR7 promote metastasis by preventing anoikis in cancer cells. Cell Death Differ 16: 664–673. 10.1038/cdd.2008.190 19136936

[34] Hasegawa M, Kamada N, Jiao Y, Liu MZ, Nunez G, Inohara N (2012) Protective role of commensals against Clostridium difficile infection via an IL-1beta-mediated positive-feedback loop. J Immunol 189: 3085–3091. 10.4049/jimmunol.1200821 22888139 PMC3752782

[35] Wang L, Wang YC, Hu XB, Zhang BF, Dou GR, He F, et al (2009) Notch-RBP-J signaling regulates the mobilization and function of endothelial progenitor cells by dynamic modulation of CXCR4 expression in mice. PLoS One 4: e7572 10.1371/journal.pone.0007572 19859544 PMC2762521

[36] Li L, Tan J, Zhang Y, Han N, Di X, Xiao T, et al (2014) DLK1 Promotes Lung Cancer Cell Invasion through Upregulation of MMP9 Expression Depending on Notch Signaling. PLoS One 9: e91509 10.1371/journal.pone.0091509 24621612 PMC3951400

[37] Weber A, Wasiliew P, Kracht M (2010) Interleukin-1 (IL-1) pathway. Sci Signal 3: cm1. 20086235 10.1126/scisignal.3105cm1

[38] Elango KJ, Suresh A, Erode EM, Subhadradevi L, Ravindran HK, Iyer SK, et al (2011) Role of human papilloma virus in oral tongue squamous cell carcinoma. Asian Pac J Cancer Prev 12: 889–896. 21790221

[39] Gonzalez-Losa Mdel R, Canul-Canche J, Calderon-Rocher C (2009) Human papillomavirus 58 in a squamous cell carcinoma of the tongue. Oral Oncol 45: e72 10.1016/j.oraloncology.2009.02.002 19362048

[40] Tsimplaki E, Argyri E, Xesfyngi D, Daskalopoulou D, Stravopodis DJ, Panotopoulou E (2014) Prevalence and expression of human papillomavirus in 53 patients with oral tongue squamous cell carcinoma. Anticancer Res 34: 1021–1025. 24511049

[41] Nordfors C, Grun N, Tertipis N, Ahrlund-Richter A, Haeggblom L, Sivars L, et al (2013) CD8+ and CD4+ tumour infiltrating lymphocytes in relation to human papillomavirus status and clinical outcome in tonsillar and base of tongue squamous cell carcinoma. Eur J Cancer 49: 2522–2530. 10.1016/j.ejca.2013.03.019 23571147

[42] Wang N, Feng Y, Wang Q, Liu S, Xiang L, Sun M, et al (2014) Neutrophils infiltration in the tongue squamous cell carcinoma and its correlation with CEACAM1 expression on tumor cells. PLoS One 9: e89991 10.1371/journal.pone.0089991 24587171 PMC3937421

[43] Kauppila JH, Mattila AE, Karttunen TJ, Salo T (20113) Toll-like receptor 5 (TLR5) expression is a novel predictive marker for recurrence and survival in squamous cell carcinoma of the tongue. Br J Cancer 108: 638–643. 10.1038/bjc.2012.589 23287987 PMC3593548

[44] Lewis AM, Varghese S, Xu H, Alexander HR (2006) Interleukin-1 and cancer progression: the emerging role of interleukin-1 receptor antagonist as a novel therapeutic agent in cancer treatment. J Transl Med 4: 48 17096856 10.1186/1479-5876-4-48 PMC1660548

[45] Muller A, Homey B, Soto H, Ge N, Catron D, Buchanan M E, et al (2001) Involvement of chemokine receptors in breast cancer metastasis. Nature 410: 50–56. 11242036 10.1038/35065016

[46] Roberts PJ, Der CJ (2007) Targeting the Raf-MEK-ERK mitogen-activated protein kinase cascade for the treatment of cancer. Oncogene 26: 3291–3310. 17496923 10.1038/sj.onc.1210422

[47] Neufert C, Becker C, Tureci O, Waldner MJ, Backert I, Floh K, et al (2013) Tumor fibroblast-derived epiregulin promotes growth of colitis-associated neoplasms through ERK. J Clin Invest 123: 1428–1443. 10.1172/JCI63748 23549083 PMC3613905

[48] Kikuchi Y, Kunita A, Iwata C, Komura D, Nishiyama T, Shimazu K, et al (2014) The niche component periostin is produced by cancer-associated fibroblasts, supporting growth of gastric cancer through ERK activation. Am J Pathol 184: 859–870. 10.1016/j.ajpath.2013.11.012 24418260

[49] Wu S, Lao XY, Sun TT, Ren LL, Kong X, Wang JL, et al (2013) Knockdown of ZFX inhibits gastric cancer cell growth in vitro and in vivo via downregulating the ERK-MAPK pathway. Cancer Lett 337: 293–300. 10.1016/j.canlet.2013.04.003 23587796

[50] Fang Z, Tang Y, Fang J, Zhou Z, Xing Z, Guo Z, et al (2013) Simvastatin inhibits renal cancer cell growth and metastasis via AKT/mTOR, ERK and JAK2/STAT3 pathway. PLoS One 8: e62823 10.1371/journal.pone.0062823 23690956 PMC3656850

[51] Renshaw J, Taylor KR, Bishop R, Valenti M, De Haven Brandon A, Gowan S, et al (2013) Dual blockade of the PI3K/AKT/mTOR (AZD8055) and RAS/MEK/ERK (AZD6244) pathways synergistically inhibits rhabdomyosarcoma cell growth in vitro and in vivo. Clin Cancer Res 19: 5940–5951. 10.1158/1078-0432.CCR-13-0850 23918606 PMC3818134

[52] Choi C, Helfman DM (2014) The Ras-ERK pathway modulates cytoskeleton organization, cell motility and lung metastasis signature genes in MDA-MB-231 LM2. Oncogene. 33: 3668–3676. 10.1038/onc.2013.341 23995792

[53] Zhang D, Li X, Yao Z, Wei C, Ning N, Li J (2014) GABAergic signaling facilitates breast cancer metastasis by promoting ERK-dependent phosphorylation. Cancer Lett. 348: 100–108. 10.1016/j.canlet.2014.03.006 24657659

[54] Marignol L, Rivera-Figueroa K, Lynch T, Hollywood D (2013) Hypoxia, notch signalling, and prostate cancer. Nat Rev Urol 10: 405–413. 10.1038/nrurol.2013.110 23712204 PMC5240418

[55] South AP, Cho RJ, Aster JC (2012) The double-edged sword of Notch signaling in cancer. Semin Cell Dev Biol 23: 458–464. 10.1016/j.semcdb.2012.01.017 22309843 PMC3360804

[56] Asnaghi L, Ebrahimi KB, Schreck KC, Bar EE, Coonfield ML, Bell WR, et al (2012) Notch signaling promotes growth and invasion in uveal melanoma. Clin Cancer Res 18: 654–665. 10.1158/1078-0432.CCR-11-1406 22228632 PMC4648284

[57] Purow BW, Haque RM, Noel MW, Su Q, Burdick MJ, Lee J, et al (2005) Expression of Notch-1 and its ligands, Delta-like-1 and Jagged-1, is critical for glioma cell survival and proliferation. Cancer Res 65(6):2353–2363. 15781650 10.1158/0008-5472.CAN-04-1890

[58] Suman S, Das TP, Damodaran C (2013) Silencing NOTCH signaling causes growth arrest in both breast cancer stem cells and breast cancer cells. Br J Cancer 109: 2587–2596. 10.1038/bjc.2013.642 24129237 PMC3833225

[59] Yang Y, Ahn YH, Gibbons DL, Zang Y, Lin W, Thilaganathan N, et al (2011) The Notch ligand Jagged2 promotes lung adenocarcinoma metastasis through a miR-200-dependent pathway in mice. J Clin Invest 121: 1373–1385. 10.1172/JCI42579 21403400 PMC3069760

[60] Nicolas M, Wolfer A, Raj K, Kummer JA, Mill P, van Noort M, et al (2003) Notch1 functions as a tumor suppressor in mouse skin. Nat Genet 33: 416–421. 12590261 10.1038/ng1099

[61] Proweller A, Tu L, Lepore JJ, Cheng L, Lu MM, Seykora J, et al (2006) Impaired notch signaling promotes de novo squamous cell carcinoma formation. Cancer Res 66: 7438–7444. 16885339 10.1158/0008-5472.CAN-06-0793

[62] Mirandola L, Apicella L, Colombo M, Yu Y, Berta DG, Platonova N, et al (2013) Anti-Notch treatment prevents multiple myeloma cells localization to the bone marrow via the chemokine system CXCR4/SDF-1. Leukemia 27:1558–1566. 10.1038/leu.2013.27 23354012

[63] Wang YC, Hu XB, He F, Feng F, Wang L, Li W, et al (2009) Lipopolysaccharide- induced maturation of bone marrow-derived dendritic cells is regulated by notch signaling through the up-regulation of CXCR4. J Biol Chem 284:15993–16003. 10.1074/jbc.M901144200 19357083 PMC2708893

[64] Xie J, Wang W, Si JW, Miao XY, Li JC, Wang YC, et al (2013) Notch signaling regulates CXCR4 expression and the migration of mesenchymal stem cells. Cell Immunol 281:68–75. 10.1016/j.cellimm.2013.02.001 23474530

[65] Williams CK, Segarra M, Sierra Mde L, Sainson RC, Tosato G, Harris AL (2008) Regulation of CXCR4 by the Notch ligand delta-like 4 in endothelial cells. Cancer Res 68:1889–1895. 10.1158/0008-5472.CAN-07-2181 18339870

